# Low-Field Magnetic Resonance Imaging for the Detection of Medial Meniscal Lesions in Cranial Cruciate-Deficient Stifle Joints in Dogs

**DOI:** 10.3390/ani14213097

**Published:** 2024-10-27

**Authors:** Julia Knebel, Svenja K. Wilke, Stephan Neumann, Anna-Lena Klatt, Henning C. Schenk, Martin Konar

**Affiliations:** 1 Small Animal Clinic Lueneburg, 21337 Lueneburg, Germany; s.wilke@tierklinik-lueneburg.de (S.K.W.); annalena.klatt@web.de (A.-L.K.); h.schenk@tierklinik-lueneburg.de (H.C.S.); 2 Institute of Veterinary Medicine, Georg August University, 37073 Goettingen, Germany; sneuman@gwdg.de; 3 Independent Researcher, 54100 Marina di Massa, Italy; martinkonar@outlook.com

**Keywords:** cranial cruciate ligament rupture, medial meniscal lesions, noninvasive diagnostic, low-field magnetic resonance imaging, dog

## Abstract

Background: The aim of this study was to substantiate the ability of noninvasive low-field magnetic resonance imaging (lfMRI) to accurately depict meniscal lesions, thereby establishing lfMRI as a viable option for the diagnosis of meniscal pathology. Study Design: This was a prospective, controlled study. Materials and Methods: In each of 57 stifle joints, 5 predefined lfMRI sequences using a 0.25 T lfMRI were used. The scans were evaluated by an experienced surgeon (DECVS) and through external assessment by a radiologist (DECVDI). The patients were divided into two groups depending on their meniscal status after lfMRI. Dogs (Group 1; *n* = 33) without lfMRI evidence of medial meniscal pathology underwent TPLO, whereas concurrent craniomedial mini-arthrotomy was performed in patients with suspected meniscal lesions (Group 2; *n* = 24). Gait assessment was performed 6 weeks later and 6 months postoperatively. Results: Cohen’s kappa value of 0.8571 corresponds to a high level of agreement between the observers. A significant correlation was found between preoperative lameness severity and intraoperative meniscal damage (*p* < 0.04). The results showed a highly significant correlation between meniscal injuries on MRI and intraoperative findings (*p* < 0.0001). Conclusions: LfMRI appears to be a noninvasive, reliable imaging tool for the detection of medial meniscal lesions in cranial cruciate-deficient stifle joints, aiming to avoid (minimally) invasive diagnostics in healthy menisci.

## Simple Summary

Magnetic resonance imaging (MRI) is a noninvasive diagnostic modality for assessing cranial cruciate ligament (CCL) deficiency in stifles, particularly for identifying medial meniscal lesions (MML). In cases of canine meniscal injury, mostly following cruciate ligament tears, detecting meniscal damage is crucial for appropriate patient management. In small animals, arthroscopy is considered the gold standard for diagnosing meniscal tears, as it allows direct visualization of the meniscus and the possibility of surgical intervention if needed, but it is not noninvasive. To assess the usefulness of low-field MRI (lfMRI) in accurately depicting treatable meniscal pathologies and to avoid invasive intra-articular diagnostics in healthy menisci, 57 stifle joints of dogs with cranial cruciate ligament deficiency were evaluated. Finally, lfMRI was viewed as a feasible, noninvasive imaging tool for the assessment of the medial meniscus in cranial cruciate deficient stifle joints.

## 1. Introduction

Arthroscopy and magnetic resonance imaging (MRI) are two diagnostic tools used in the evaluation of cranial cruciate ligament (CCL)-deficient stifles, specifically for the detection of medial meniscal lesions (MML) [1]. Canine meniscal injury follows cruciate ligament tears, and the identification of meniscal damage is crucial for guiding patient management [2]. MMLs appear either as primary injuries mostly associated with cranial cruciate ligament insufficiency or as secondary lesions after previous stabilization [3].

The number of MMLs diagnosed varies between 10% and 70% [4]. The prevalence of late meniscal injuries is reported to be between 2.8% and 20% [5,6,7,8]. During orthopedic examination, a so-called “meniscal click” can be heard or felt as an indication of a meniscal lesion. However, it is a less sensitive test, with only 27% to 63% of patients with MMLs experiencing a “meniscal click” [6,9]. Additional diagnostic options for meniscal injuries include noninvasive techniques such as magnetic resonance imaging (MRI), computed tomography (CT), and ultrasound. High-field MRI allows reliable meniscal diagnostics with a sensitivity of 100% and specificity of 96% [10,11]. Arthroscopy, in contrast, is considered to be the gold standard [6] for the diagnosis of meniscal tears because it allows direct visualization of the meniscus and the ability to perform surgical intervention if necessary [12,13]. In the context of arthroscopy, the sensitivity for diagnosing MML can be increased from 60% to 85% by using a joint distractor. In the study by Winkels et al. [14], the diagnosis was improved 1.9 times by using the Leipzig joint distractor. Similarly, the specificity of arthroscopy can increase from 92% to 96% due to good visualization of the medial meniscus [15,16]. Additionally, the use of a probe allows the correct visualization of meniscal damage in more than 90% of cases, and the accuracy of diagnosis increases eightfold [17]. In a comparative study of MRI and arthroscopy, Crues et al. [18] reported a sensitivity and specificity of approximately 92% for meniscal lesions. Both techniques can identify exactly the same locations of meniscal lesions [19]. In another study, the sensitivity of lfMRI was 0.64 (95% confidence (95% CI) = 0.43, 0.80) and the specificity was 0.90 (95% CO = 0.70, 0.97). Finally, the authors concluded that low-field MRI has a low diagnostic accuracy for detecting MML, so the risk of overlooked tears must be considered [12]. Compared to that of lfMRI, the sensitivity of high-field MRI for the detection of meniscopathies is as high as 90%, with a specificity of 96% [10]. However, the sensitivity of both arthroscopy and MRI can be affected by factors such as the experience of the operator [13], the quality of the equipment, the chosen MRI sequence [11], and the size and location of the meniscal tear [1].

The aim of this study was to determine the sensitivity and specificity of lfMRI for diagnosing surgically treatable MML in cranial cruciate-ligament-deficient stifle joints in medium- and large-breed dogs. The authors hypothesized concordance between the reviewers and the comparison to the intraoperative findings.

## 2. Materials and Methods

### 2.1. Study Design

This prospective, randomized, controlled study included 57 stifle joints of 55 dogs with cranial cruciate ligament deficiency that presented at the Small Animal Clinic, Lueneburg, from June 2021 to February 2022. The criteria for dogs to be included in the study were a bodyweight between 20 and 53 kg and the absence of other serious systemic diseases. In addition to obtaining a history of lameness duration, a general and clinical orthopedic examination was conducted preoperatively for all patients. The subjective assessment of gait was classified into six grades (grades 0–5). The palpatory examination of the stifles included assessment of joint effusion; reproducible pain on extension, flexion, and rotation (painful/nonpainful); the presence of cranial tibial thrust via cranial drawer and tibial compression tests (positive/negative); verification of intra-articular crepitus (grade 0–3); and the presence of a “meniscal click” (present/absent). The evaluation of femorotibial instability, as well as the elicitation of a meniscal click, was repeated under anesthesia for all joints. Dogs who underwent lfMRI of the affected stifle joint were divided into two groups depending on their meniscal status. Dogs (Group 1; *n* = 33) without lfMRI evidence of medial meniscal pathology received tibia plateau leveling osteotomy (TPLO), whereas concurrent craniomedial mini-arthrotomy and partial medial meniscectomy were performed in patients with suspected meniscal lesions (Group 2; *n* = 24). Radiographs in mediolateral and craniocaudal views were taken of each patient and stifle joint before and after surgery, as well as during re-evaluation. The owners of the dogs were interviewed 6 weeks and 6 months postoperatively for evidence of complications. Major complications requiring revision surgery were assessed over the study period.

### 2.2. Diagnostic and Surgical Procedures

The dogs received a standard anesthetic protocol for both techniques. Radiographs were taken preoperatively in two orthogonal views and special TPLO views [20]. For meniscal evaluation, lfMRI was performed with a 0.25 Tesla (T) system (ESAOTE Vet-MR GRANDE, Genova, Italy). All dogs were placed in lateral recumbency with the affected stifle up and the joints at a physiological angle of approximately 130° between the femur and tibia. A DPA Coil—Small Animals Coil 4 (ESAOTE, Genova, Italy) was used as a standard. Initially, 4 scout MRI scans, prescout and scout in transverse, dorsal, and sagittal positions, were obtained. Thereafter, the stifles were evaluated on 5 predefined sequences: fast short tau inversion recovery sequence (STIR) dorsal, gradient echo T2 star sequence (GE T2*) dorsal, GE T2* sagittal, 3D steady-state T1-weighted sequence (3D SST1) sagittal, and fast spin echo T2-weighted sequence (FSE T2) transverse. The scan parameters used depended on the sequences and are shown in Table 1. The lfMRI images were evaluated by the surgeon (DECVS) immediately following diagnostic imaging and preoperatively. Furthermore, an additional independent assessment of the images was conducted by an external radiologist (DECDI) who was blinded to the results of the patient’s medical history and orthopedic examination. The interpretation of the stifle joint structures was conducted according to a predefined scheme, with any pathological changes graded according to severity. The assessments included cruciate ligaments, collateral ligaments, patellar ligaments, joint capsules, joint effusion, osteophytes, cartilage, and menisci, with a specific emphasis on meniscal evaluation in this study (Table 2). The evaluation of the menisci involved considering various factors across different sequences, with the GE T2* (both dorsal and sagittal) being most relevant for meniscal interpretation. A healthy meniscus appeared as a hypointense structure in these MRI images, with triangular-shaped anterior and posterior horns evident in sagittal sections. The absence of these characteristic triangular structures suggested meniscal pathology. Meniscal tears were identified by an increase in signal, morphological changes, and potential fragment formation, with or without displacement. Defects were further visualized as signal enhancement at the location of missing meniscal tissue. The diagnosis of meniscal pathologies guided the decision-making process regarding the necessity of craniomedial mini-arthrotomy. In the cases of mini-arthrotomy, an intraoperative description of the pathological changes was conducted to compare the findings with the MRI findings. After diagnostic imaging, the affected stifle was clipped and aseptically prepared for surgery. The DECVS performed all surgical procedures. The TPLO was performed according to the procedure described by Slocum and Slocum [20]. In cases of a diagnosed MML, a craniomedial mini-arthrotomy was performed prior to TPLO through the same approach to partially resect the injured part of the medial meniscus [21]. The type of meniscal lesion was described intraoperatively based on Beale’s meniscal classification scheme (Table 3) [22]. A grading system ranging from 0 to 7 was utilized, wherein grade 0 represents a completely intact, unaltered meniscus, while grade 7 encompasses multiple meniscal lesions. After both procedures, radiographs of the affected stifle were obtained in two orthogonal planes to evaluate the osteotomy and implant positioning. All patients received the same pain and antibiotic medication, which consisted of cefalexin (25 mg/kg BID for 7 days) and meloxicam (0.1 mg/kg SID for 14 days). All patients received a bandage for 24 h and were discharged from the hospital on the day of surgery.

### 2.3. Data Analysis

Statistical analysis was performed using GraphPad Prism version 9. Cohen’s kappa test was used for the statistical calculations of interobserver variability. This measure considers both the agreement that could occur by chance and the agreement observed between the different observers. A Cohen’s kappa value of approximately 1 indicates a strong degree of agreement that goes beyond what would be expected by chance alone. In addition, the McNemar test was employed for the comparative analysis of preoperative MRI assessments and intraoperative findings, considering the latter the gold standard. The McNemar test is a statistical method specifically designed for assessing differences in paired categorical data. Additionally, the Spearman rank correlation coefficient was used to determine the degree of association between the two assessment methods. In addition, the predictability of lameness severity after 6 weeks or 6 months following the preoperative evaluation of the medial meniscus was investigated. The analysis was conducted using a repeated-measures ANOVA to evaluate differences in lameness grades across different time points. The relationship between the severity of lameness before surgery and the presence of meniscal damage was evaluated. Spearman’s rank correlation coefficient was used to determine the degree of association between the ordinal variables. For all tests, significance was set at 5% (*p* ≤ 0.05).

## 3. Results

### 3.1. Signalment and Descriptive Statistics

LfMRI was performed in a total of 57 stifles of 55 dogs with cranial cruciate ligament pathology. The mean body weight recorded was 34 kg (range 20–53 kg), and the average age was 4.5 years (range 1–11 years). The dominant breeds in this study were Golden Retrievers (*n* = 8), American Staffordshire Terriers (*n* = 6), and Bulldogs (*n* = 11).

### 3.2. Clinical and Orthopedic Examination

Physical and orthopedic examination revealed moderate (grade 2 + 3; 51%) to severe (grade 4 + 5; 49%) hindlimb lameness, increased stifle joint effusion (100%), pain during extension (100%), and stifle joint instability during assessment under anesthesia, as indicated by a positive cranial drawer sign (89.0%) or cranial tibial thrust (95%). A total of 27 right hindlimbs and 31 left hindlimbs were affected. Bilateral pathology was present in two dogs.

### 3.3. Diagnostic and Surgical Procedures

**Figure 1:**
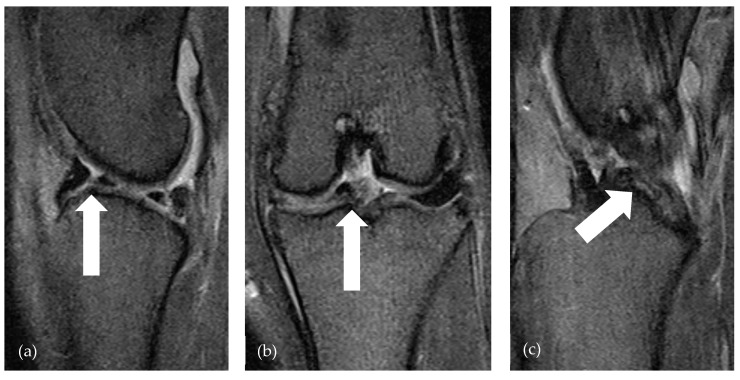
lfMRI of bucket handle tear (arrows) of medial meniscus in GE T2* sag (**a**), GE T2* dor (**b**), and GE T2* sag (**c**). The arrow in (**a**) points to the fragment formation, the arrow in (**b**) points to the axial displaced fragment, and the arrow in (**c**) shows the fragment just cranial to the caudal cruciate ligament.

Preoperative radiographic assessments acquired under anesthesia revealed the presence of an increase in the soft tissue opacity associated with distension of the stifle joint across all patients. Postoperative radiographic assessments indicated proper positioning of all implants. The total duration of the MRI examination, encompassing the localizer, sequence planning, and 3D reconstruction, was 31.57 min. The examination may take a maximum of 1 min longer for dogs over 40 kg bodyweight due to the mass differences of the stifle joints in large dogs. There was no significant difference (*p* = 0.6831) between the MRI findings and the modified intraoperative screening according to Beale’s meniscal classification [23] in the evaluation of the medial meniscus via lfMRI and the intraoperative examination. The results showed a highly significant correlation between meniscal injuries on MRI and intraoperative findings (Spearman’s Rho = 0.8, *p* < 0.0001). In 19/19 patients, a bucket-handle tear of the medial meniscus, which was diagnosed via lfMRI, was confirmed intraoperatively. Notably, a substantial proportion of dogs with medial meniscal pathology exhibited severe alterations of grade 5 (*n* = 22 out of 25) (Figure 1). Regarding the interobserver variability, there was significant agreement between the observers in the interpretation of 14 stifle joint structures, with a specific emphasis on meniscal evaluation in the MRI sequences, as there were no significant differences (*p* > 0.05) between the individual assessments in 13 out of 14 structures. Therefore, Cohen’s kappa was 0.8571. This corresponds to a high level of agreement between the observers. When considering only the interobserver agreement regarding the assessment of the medial meniscus, there is also no significant difference (*p* = 0.70). A significant correlation was found between preoperative lameness severity and intraoperative meniscal damage (*p* < 0.04). This suggests that patients with greater preoperative lameness severity are significantly more likely to experience intraoperative meniscal damage (Table 4).

### 3.4. Evaluation 6 Weeks and 6 Months Postoperatively

**Figure 2:**
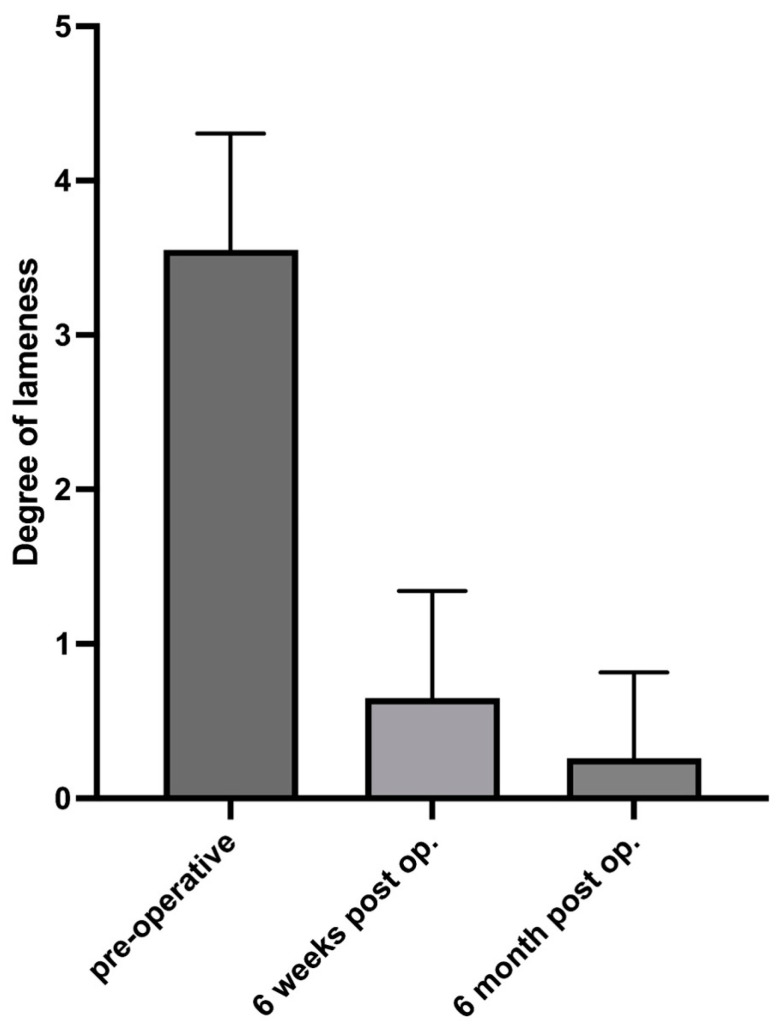
Lameness evaluation preoperatively and at 6 weeks and 6 months postoperatively.

In this study, the severity of lameness in 51/57 stifle joints was assessed clinically 6 weeks after surgery. Fifty-one owners were interviewed via telephone 6 months after surgery regarding the gait of their dogs and any complications during the study period. One dog was euthanized, which was unrelated to the orthopedic conditions. The results showed a highly significant effect of time on lameness severity (F = 404, *p* < 0.001), indicating a significant change in lameness severity over time, with an F value of 404 suggesting that this difference is extremely significant. Post hoc analysis revealed that lameness grades improved significantly from preoperative values to 6 weeks and 6 months post-surgery (*p* < 0.001). Lameness severity at 6 months post-surgery was significantly lower than that at 6 weeks post-surgery (*p* < 0.001) (Figure 2). These results suggest a significant improvement in lameness severity during the postoperative recovery period, demonstrating that there was no evidence of missed meniscal tears or subsequent postoperative tears in study dogs. A *p* value of less than 0.001 indicates that the observed differences are highly unlikely to be due to random variation. No major complications that required revision surgery occurred in any of the treated dogs.

## 4. Discussion

In this study, 57 stifle joints with (partial) rupture of the cranial cruciate ligament were examined by lfMRI to assess the reliability and reproducibility of this technique for diagnosing surgically treatable MML. MRI is a noninvasive imaging modality that uses magnetic fields and radio waves to produce detailed information about the morphology and location of meniscal tears without the need for surgery [10,11,18]. In the present study, it was demonstrated that the lfMRI of the medial meniscus and the subsequent classification into lesions requiring surgical revision and those not requiring surgical therapy is reliable.

### 4.1. Meniscal Evaluation

**Figure 3:**
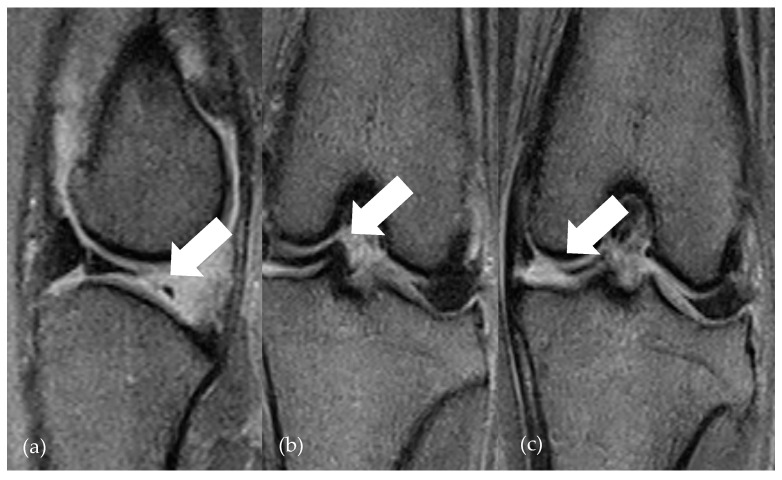
lfMRI of a folded fragment (arrows) of medial meniscus in GE T2 sag (**a**) and GE T2 dor (**b**,**c**). The arrow in (**a**) points to the missed meniscal tissue, the arrow in (**b**) points to the axial displaced fragment, and the arrow in (**c**) shows the reproducible increase in signal in the dorsal view.

Thus, the highly significant correlation between meniscal evaluation on lfMRI and intraoperative findings indicates that the findings on MRI closely correlate with intraoperative findings. Furthermore, there was a significant improvement in lameness severity within the study period without indication of persistent or secondary meniscal injury during the study period. Regarding the clinical examinations, it is assumed that there are more severe lameness grades in cases of additional meniscal injury in the cruciate-ligament-insufficient knee joint [24]. This was also observed in this study, where dogs with preoperative magnetic resonance imaging evidence of MML exhibited more pronounced lameness. Consequently, this correlation underscores the correct interpretation of meniscal evaluation and the appropriate assessment of nontreatable medial menisci in patients of Group 2 can be concluded. This statement contrasts with the conclusion drawn by Böttcher et al. [12], who postulated the increased risk of both false-positive and false-negative meniscal injuries compared to arthroscopy. Only 14/22 meniscal alterations were identified by lfMRI. An optimization of accurate meniscal diagnostics allows for the utilization of high-field MRI. Blond et al. [11,13] describe a sensitivity of 100% and a specificity of 94% in diagnosing meniscal injuries with a 1.5 T MRI. Regardless of technical conditions, however, the use of a standardized MRI protocol is helpful in improving the reliability of MRI-based meniscal diagnostics [1]. In this study, examinations were conducted following a standardized procedure and protocol. Furthermore, the use of an established schema facilitates the comprehensive and reproducible assessment of various stifle joint structures. Thus, no significant difference was found between a radiologist specialized in MRI examinations and an experienced surgeon regarding accurate meniscal diagnostics in the present study. The more reliable interpretation of the meniscus in the context of lfMRI compared to previous studies can likely be attributed to the improved technology of the devices as well as the greater experience of surgeons and radiologists in image interpretation. Martig et al. [1] have already recommended the use of an established protocol to optimize results. Böttcher et al. also criticized observer-dependent interpretation. Since no significant correlation between the observer’s experience and sensitivity or specificity could be found, there is a desire for reproducible lfMRI criteria for meniscal diagnostics [13]. The observers in the present study differed in their training, experience, and specialization (DECVS and DECVDI). Presumably due to the use of the interpretation scheme, significant agreement between the observers was observed. Nevertheless, the specific evaluation of the menisci involves consideration of various factors across the sequences. The most prevalent form of meniscal pathology, the bucket handle tear, manifests as a longitudinal vertical tear, with or without medial displacement of the free part. In the transverse section, visualization of the bucket handle tear alongside the remaining intact meniscal part is feasible [25]. A further improvement in specificity of lfMRI in diagnosing meniscal pathologies can be achieved by transverse reformatting 3D sequences (Figure 3). Further studies are needed to test the superiority of this additional sequence.

### 4.2. Minimally Invasive Versus Noninvasive Diagnostic Imaging

In the literature, diagnostic arthroscopy is considered to be the gold standard for diagnosing meniscal pathologies. Not only optimal visualization but also time effective feasibility is frequently regarded positively. The duration of noninvasive lfMRI diagnostics of the stifle joint is 32 min in the present study. Whether this timeframe represents a significant difference compared to minimally invasive arthroscopy, including patient positioning and setup, needs further investigation through additional studies. The fact remains that noninvasive diagnostics such as lfMRI eliminate the need for stifle joint opening in the absence of evidence of meniscal lesions. Especially in patients without indications of meniscal pathology, avoiding (minimally) invasive diagnostics involving joint opening is a noteworthy advantage of noninvasive MRI. This approach may help reduce the risks of increased postoperative morbidity, surgery-related complications, and osteoarthritis progression [26,27,28,29,30]. For instance, arthroscopy is known to be the gold standard for detecting MMLs compared to MRI; nevertheless, both modalities have their own advantages and limitations [17]. The choice of diagnostic tool should be based on the individual patient needs, the suspected pathology, and the available resources. Limitations include the fact that the status of those menisci, which were classified as healthy on lfMRI, was not specified through mini-arthrotomy. To avoid unnecessary, even minimally invasive, arthroscopic access to the joint, patients were interviewed or clinically evaluated by the owners over a period of 6 months. The absence of any indication of overlooked meniscal lesions led to the assumption that adequate assessment had taken place. Furthermore, while the sensitivity and specificity of mini-arthrotomy used to confirm pathological findings can be improved using a probe, it does not reach the success rate of arthroscopic evaluation [17]. However, the advantage of cranial medial mini-arthrotomy lies in the use of a minimally extended approach combined with TPLO and minimal joint access. To what extent the use of MRI versus arthroscopic diagnosis for evaluating meniscal pathologies before performing TPLO is superior in terms of postoperative morbidity or progression of degenerative joint changes can only be speculated upon and has to be proven based on further studies. However, it is apparent that the increasing availability of MRI, along with the use of reproducible protocols and assessment schemes, as well as growing experience in interpretation, supports subsequent appropriate therapy and avoids unnecessary joint access.

## 5. Conclusions

Finally, LfMRI is a noninvasive, reliable imaging tool for the assessment of medial meniscal pathologies in cranial cruciate-deficient stifle joints that avoids (minimally) invasive diagnostics.

## Figures and Tables

**Table 1:** LfMRI stifle joint protocol.

Sequence	Slice Thickness/ Slice Separation	Time to Repeat (TR)	Time to Echo (TE)	Flip Angle (FA)	Acquisition Time
Prescout					0:13 min
Scout transversal	4.0/0.4 mm				0:41 min
Scout dorsal	4.0/0.4 mm				0:40 min
Scout sagittal	4.0/0.4 mm				0:40 min
Fast Stir dorsal	3.0/0.3 mm	4220 ms	30 ms	90°	5:46 min
GE T2* dorsal	2.5/0.2 mm	1675 ms	26 ms	65°	6:24 min
GE T2* sagittal	2.5/0.2 mm	1675 ms	26 ms	65°	6:24 min
FSE T2 transversal	3.0/0.2 mm	7220 ms	120 ms	90°	6:31 min
3D SST1 sagittal	0,35 mm in 3D	22 ms	9 ms	30°	4:15 min
Total acquisition time					31:57 min

**Table 2:** LfMRI grading system for the medial and lateral meniscus.

Grade	Meniscal Pathology
0	without pathological findings
1	diffusely increased signal—degeneration
2	mild axial fibrillation; mildly irregular axial border
3	severe axial fibrillation; blunt axial border without presence of fragments
4	nondisplaced rupture (linear signal increase with contact to one or two surfaces)
5	rupture with displaced fragment, bucket handle tear
6	folded meniscus
7	large parts of meniscus missing, multiple fragments

**Table 3:** Grading system intraoperative (in addition to Beale et al. 2003 [23]).

Grade	Meniscal Pathology
0	without pathological findings
1	meniscus degeneration
2	mild axial fraying
3	severe axial fraying
4	not displaced rupture
5	bucket handle tear
6	folded medial meniscus
7	multiple dislocated parts of meniscus

**Table 4:** Preoperative lameness grade (0–5), lfMRI interpretation of the medial meniscus (grade 0–7, Table 2) by the first and second observer, as well as intraoperative meniscus findings (grade 0–7, Table 3) in dogs with meniscal lesions (Group 2).

Patient	Lameness Preoperative	lfMRI Observer 1	fMRI Observer 2	Grading Intraoperative
1	4	5	5	5
2	3	5	5	5
3	5	5	0	5
4	4	5	7	5
5	4	3	3	4
6	5	5	5	5
7	5	0	0	0
8	3	5	5	5
9	5	5	6	5
10	4	5	5	5
11	3	3	0	3
12	4	5	5	5
13	3	5	3	5
14	4	4	1	4
15	4	5	5	5
16	4	5	5	5
17	3	5	5	5
18	5	5	5	5
19	3	5	5	5
20	5	5	5	5
21	3	5	5	5
22	3	7	5	6
23	4	5	5	5
24	4	7	5	7

## Data Availability

No new data are available.
